# Peer Review of “Impact of Weekly Community-Based Dance Training Over 8 Months on Depression and Blood Oxygen Level–Dependent Signals in the Subcallosal Cingulate Gyrus for People With Parkinson Disease: Observational Study”

**DOI:** 10.2196/67811

**Published:** 2024-12-13

**Authors:** Anonymous

**Keywords:** depression, cingulate, GDS, Geriatric Depression Scale, neuroimaging, dancing, Parkinson disease, neurodegenerative, MRI, imaging, neurology, magnetic resonance imaging

*This is the peer-review report for “Impact of Weekly Community-Based Dance Training Over 8 Months on Depression and Blood Oxygen Level–Dependent Signals in the Subcallosal Cingulate Gyrus for People With Parkinson Disease: Observational Study.”*

## Round 1 Review

### General Comments

I thank the editors for the opportunity to review this article titled “Impact of Weekly Community-Based Dance Training Over 8 Months on Depression and Blood Oxygen Level–Dependent Signals in the Subcallosal Cingulate Gyrus for People With Parkinson Disease: Observational Study” [1]. In this article, the authors report a challenging and well-designed study into the effects of an 8-month dance program designed specifically for reducing nonmotor symptoms in individuals with Parkinson disease on behavioral measures and functional magnetic resonance imaging (fMRI) responses. The article reviews the literature around nonmotor symptoms and the treatment thereof in individuals with Parkinson disease, and the limited existing evidence around the mechanisms of action of these treatments. The authors address the lack of larger-scale studies showing the benefits of dance therapy in this population. The article concludes that dance therapy provides a promising treatment option for nonmotor symptoms in people with Parkinson disease.

Below are some comments that the authors may wish to integrate into future revisions of their work.

### Specific Comments

#### Major Comments

1. My main concerns are around the description of the methods used in the study. There is generally not enough detail or justification for decisions made in the acquisition and analysis of the data presented.

2. At the end of the introduction, the “SCG” (subcallosal cingulate gyrus) is mentioned but without further context. As the main finding of the paper rests on the use of the SCG as a region of interest, it would be good to understand more about why only this area was investigated. Were other areas explored/analyzed? If it was the intention to look only at the SCG, why was the field of view so large in the fMRI acquisition? If other areas were looked at, these analyses need to be included. If it was the intention to only look at the SCG, more justification needs to be given as to why this was the only area investigated in the dataset.

3. I acknowledge that the sample sizes in the existing literature are of the order n=1, and that a sample of n=10 is a significant improvement on this. However, the description of the sample sizes at each stage of fMRI acquisition is somewhat confused. Did you present the analysis of the healthy control data? What was this used for? Did you present the analysis of the remaining 7 individuals who only completed 1 scan session? How many completed scanning at the baseline session? Perhaps a table or something would be useful to elucidate these numbers less ambiguously.

4. Head motion: could a quantitative comparison be made between the amount of head motion in the people with Parkinson disease group compared to controls? The methods state that no “obvious” motion artifacts were present and that no scans from the people with Parkinson disease group were removed—how was this determined? Was there an objective threshold for what would be excluded? Was motion correction used (and therefore, in what software)? Were any images removed due to motion from the control group?

5. The 30-second “OFF” period seems short and poorly described. What measures were taken to ensure that the participant stopped thinking about the dance or hearing the music playing in their head? Were they instructed to perform another task? How do you know you have not just found that listening to music with positive meaning reduces activity in this region? Was there at least a fixation cross? Please clarify and elaborate on why this is an appropriate design.

6. There are several points in the *Results* section where methods are presented (eg, first paragraph of the *Results* section). Please move all the descriptions of methods into the *Methods* section and please organize this more logically into behavioral methods (acquisition and analysis) and MRI measures (acquisition, preprocessing, and analysis). No statistical methods are described in the *Statistical Analysis* section. Please describe here what statistical tests you used (*t* tests or analysis of covariance?). You state that “no significant interaction was found between experience and GDS”—what test was used? Please outline all statistical tests conducted in the relevant *Statistical Analysis* section of the *Methods* section. Which time point was used to determine this? Beginning versus end? Please describe exactly what was done.

7. Figure 1: please show which comparisons were significant using asterisks and *P* values.

8. Figure 2: I still do not understand the sample sizes used in each of the analyses. Why is B only referring to n=7 people with Parkinson disease—surely you have n=23 for the Geriatric Depression Scale (GDS) data? Then C refers to n=10. This may be resolved by more clearly describing what data were collected in the *Methods* section as I have requested earlier. But please also display clearly in the format “n=?” on each part of the figure and in the caption how many people were included in that analysis and make clearer throughout why this number is used.

#### Minor Comments

9. In order to fully introduce dance therapy, it would be useful if the authors could refer to and cite some more work assessing the effects of dance therapy on other conditions—I feel that there is a wider bank of evidence for its efficacy in mood disorders and a wider bank of evidence that would give the introduction a more compelling context.

10. Paragraph 3 of the *Introduction*: “QoL” is mentioned—can the authors briefly add reference to the quality of life measure(s) used in these studies?

11. Just above Figure 1, you refer to a previous publication reporting Berg Balance Scale and Timed Up and Go analysis—can you (in the *Discussion*) compare the effect size reported with the effect size seen in this study?

12. It is a tiny point, but please refer to an “MR scanner,” not an “MRI scanner.”

## Abbreviations

fMRI: functional magnetic resonance imaging
GDS: Geriatric Depression Scale
SCG: subcallosal cingulate gyrus
